# Toxoplasmosis and mental disorders in the Russian Federation (with special reference to schizophrenia)

**DOI:** 10.1371/journal.pone.0219454

**Published:** 2019-07-10

**Authors:** Ekaterina V. Stepanova, Anatoly V. Kondrashin, Vladimir P. Sergiev, Lola F. Morozova, Natalia A. Turbabina, Maria S. Maksimova, Dmitry V. Romanov, Marina A. Kinkulkina, Alena V. Lazareva, Evgeny N. Morozov

**Affiliations:** 1 Martsinovsky Institute of Medical Parasitology, Tropical and Vector-Borne Diseases, I.M. Sechenov First Moscow State Medical University, Moscow, Russian Federation; 2 Department of Tropical Medicine and Parasitic Diseases, I.M. Sechenov First Moscow State Medical University, Moscow, Russian Federation; 3 Department of Psychiatry & Psychosomatics, I.M. Sechenov First Moscow State Medical University, Moscow, Russian Federation; 4 Mental Health Research Center, Moscow, Russian Federation; 5 Department of Psychiatry & Substance Addiction, I.M. Sechenov First Moscow State Medical University, Moscow, Russian Federation; 6 Department of Tropical, Parasitic Diseases and Disinfectology, Russian Medical Academy of Continuous Professional Education, Moscow, Russian Federation; Taipei Medical University/Medicine, TAIWAN

## Abstract

The association of latent toxoplasmosis with mental disorders in general and with schizophrenia in particular was noticed in the mid-1950s. In subsequent years, the role of *Toxoplasma gondii* was established based on its ability to survive for long periods of time in the nerve cells of the brain. The acute manifestations of the infection include psychopathic symptoms resembling those of schizophrenia. In the former USSR, and in other parts of the world, a number of studies were performed with respect to the association of latent toxoplasmosis and schizophrenia. However, with the dissolution of the USSR at the beginning of the 1990s, studies on the subject were halted due to financial problems and have resumed only recently. The reasons for the resumption of such studies in contemporary Russia are related to the progressively increasing incidence of schizophrenia over the last 25–30 years in the country. According to official data, approximately 550 000 persons reported suffering from the disease in 2014. There are reasons to believe that this is only a fraction of the real burden of the disease. Economically, it cost the state no less than approximately US $10 billion. The purpose of the study was to identify the level of toxoplasmosis seroprevalence in patients with verified diagnoses of schizophrenia in comparison to healthy people in Moscow City and in the Moscow region. A total of 155 persons constituted the patients group and 152 healthy people were in the control group. An integrated approach to the diagnosis and comparison of data from the entire spectrum of serological markers of infection was used, including the detection of specific IgM and the determination of IgG concentrations. It was found that among persons with neuropsychiatric disorders, the incidence of cases with latent toxoplasmosis was higher than in the control group. The effect of toxoplasmosis was significant and similar for men and women. Further statistical analyses revealed that among patients with a diagnosis of schizophrenia, the incidence of latent toxoplasmosis was significantly higher than in the control group. These data are in agreement with the results of similar studies in other countries.

## Introduction

The causative agent of toxoplasmosis–*Toxoplasma gondii* Nicolle et Manceux–was discovered in 1909. The definitive hosts of *T*. *gondii* are different species of Felidae. *T*. *gondii* is one of the world’s most pervasive parasites, infecting a wide range of mammals, including humans, that act as intermediate hosts [1, 2]. *Toxoplasma* is an intracellular parasite and can affect different cells of mammals. These protozoa are neurotropic agents [3, 4].

Two types of toxoplasmosis in humans are known: the congenital form, the result of prenatal infection from a recently infected mother, and postnatal acquired toxoplasmosis. Postnatal acquired toxoplasmosis has two forms–the transient acute toxoplasmosis, which is characterized by numerous specific and non-specific symptoms, and life-long latent toxoplasmosis, which is asymptomatic from the clinical point of view in immunocompetent subjects. The first results from infection during the prenatal period of life, and the second is contracted during the postnatal period. Rarely, certain symptoms of acute toxoplasmosis persist for months and even years as so-called chronic toxoplasmosis [5].

Chronic or latent forms are more prevalent in humans than acute forms. Toxoplasmosis constitutes a serious health problem that is responsible for a prolonged disease course with polymorphic clinical manifestations, particularly with respect to the brain (mental disorders), eyes, heart muscles and other organs [6, 7]. The prevalence of latent toxoplasmosis varies widely in different countries of the world, reaching approximately 90% in certain areas. The seroprevalence of infection is 25%-30%, on average, in Western and Eastern Europe. In the United States, an overall seroprevalence varied between 11% and 22.5% [8]. The prevalence of latent toxoplasmosis in the territory of the former USSR (until dissolution in 1991) was in the range of 10%-15%.

The results of a recent study on latent toxoplasmosis among residents of Moscow City and the Moscow region revealed that seroprevalence was 25.3% [9].

The last 15–20 years have seen a sharp increase in publications from different parts of the world on the association of latent toxoplasmosis and mental disorders [10–12]. The reason for such interest is the ability of *Toxoplasma gondii* to survive for long periods of time in the nerve cells of the brain. Acute toxoplasmosis sometimes leads to symptoms similar to those of schizophrenia, namely delusions and hallucinations [12]. Cases are known from patients with a well-verified diagnosis of acute toxoplasmosis and a primary diagnosis of schizophrenia [13–15].

The increase in production of the neurotransmitter dopamine and the hormone testosterone, as well as other *Toxoplasma*-associated changes, affects the psychomotor performance, personality profile, and behavior of infected subjects [16].

Schizophrenia is one of the most prevalent mental diseases. Positive and negative symptoms of this disease, as well as its effects on cognitive performance and behavior, have dramatic social and economic consequences [17].

The disease is characterized by a latent course with frequent remissions necessitating prolonged hospitalization, marked by increasing personality changes and feelings of invalidation. It is usually accompanied by other diseases and results in a reduction in lifespan [18].

According to the World Health Organization (WHO), there are approximately 24 million persons in the world afflicted by the disease. The highest incidence of the disease was found in Sweden (1.7%), Ireland (1.2%), Russia (0.82%), and the USA (0.72%) [19].

In the former USSR, a number of studies were performed with respect to the association of latent toxoplasmosis and mental disorders, particularly schizophrenia [20, 21]. No similar research was carried out following the dissolution of the USSR. However, the necessity to resume such studies is warranted due to the acute prevalence of schizophrenia in contemporary Russia. According to official data, approximately 550 000 persons reported suffering from schizophrenia in Russia in 2014 [22]. There are reasons to believe that these figures are only a fraction of the real burden of disease. More than 60% of mental health cases occur in individuals with disabilities, and the unemployment rate among them is at least 10-times higher than among healthy people.

As a result, the economics of the country suffer considerably from the disease burden. It costs the state alone no less than Rouble 547 billion (approximately US $10 billion as of 31 December 2017) annually [23].

The purpose of our study was two-fold. First, we aimed to identify the level of toxoplasmosis seroprevalence in a group of patients with verified schizophrenia diagnoses in comparison to a group of healthy people. Second, through the analysis of published data from the former USSR on the relationship between mental disorders and latent toxoplasmosis, we identified the prevalence during the 1960s-1990s and compared it with the present status in the Russian Federation.

## Materials and methods

The study was performed at the Sechenov First Moscow State Medical University during 2016. To establish the general prevalence of latent toxoplasmosis among residents of Moscow city, examinations were carried out on patients attending the Outpatient Department at the Clinical Center of the Sechenov First Moscow State Medical University. No special criteria were selected for examination in terms of age, sex or occupation. To address the second objective, we carried out an analytical epidemiological “case-control” study represented by two groups: patients and control. The patients group consisted of psychiatric patients with a diagnosis of schizophrenia. All members of the patients group were patients of the Psychiatry Clinic of Sechenov University. The criteria for inclusion were as follows: a) neuropsychiatric disorder diagnosis (schizophrenia); and b) aged 18–45 years. The criterion for exclusion was infectious pathologies (hepatitis, AIDS). All individuals in the control group were evaluated for the presence of a psychiatric disorder by means of a very thoroughly collected anamnesis. A total of 155 patients constituted the patients group, with 75 men and 80 women (Table 1). The control group consisted of 152 healthy persons aged 18–45 years (82 men and 70 women) who were undergoing routine medical examinations at the Clinical Centre of Sechenov University. Participants in the patients and controls groups were informed about the purposes of the study, and informed consent was obtained before enrollment in the study.

**Table 1:** Prevalence of latent toxoplasmosis in the male and female patients and controls.

Group	Patients			Controls			Odds ratio	C.I._95_	p-values
	Examined	Positive results		Examined	Positive results				
		Absolute number	Percent (%)		Absolute number	Percent (%)			
Men	75	29	39%	82	22	27%	1.72	0.88–3.37	0.11
Women	80	33	41%	70	17	24%	2.19	1.08–4.43	0.03
Total	155	62	40%	152	39	25%	1.93	1.16–3.23	0.007

Blood samples were taken from all participants of this study under sterile conditions. The samples were centrifuged at 200 g, and the sera were stored at -20°C until serological examination. All study patients were tested for the presence of IgG- and IgM-specific antibodies to *Toxoplasma gondii*. The determination of specific immunoglobulin G and M in the blood serum of the study population was performed using “Vector Toxo-IgG” (Cat. No. D-1752, Lot. No. 263) and “Vector Toxo-IgM” (Cat. No. D-1760, Lot. No. 151) enzyme-linked immunosorbent assay (ELISA) test kits (Vector-Best, Novosibirsk, Russian Federation). The concentration of IgG to *Toxoplasma gondii* in the test samples was measured in the test samples was measured in international units of IU/ml using calibration graphs.

### Serological analysis

The diagnosis of latent toxoplasmosis was based on the results of serological tests, namely on the presence and concentration of specific IgM and IgG antibodies.

A sample was considered negative if the *T*. *gondii* IgG level was under 10 IU/ml, and values above 10 IU/ml were considered positive. Evaluation of the results of the enzyme immunoassay for the determination of IgM antibodies was carried out by calculating the coefficient of positivity. The subjects with positive IgG and a negative IgM test were considered latent toxoplasmosis-positive.

The statistical significance of the results in the patients and control groups was obtained using the χ^2^ test, and the odds ratio (OR at 95% confidence interval [24]. The Statistical Package EpiInfo was employed for calculations. Additionally, we used the Pearson correlation, partial correlation, and Mantel-Haenszel common odds ratio estimate.

### Ethical considerations

The study was approved by the Research Ethics Board of Health of the Sechenov First Moscow State Medical University (protocol No. 04–13, 10.04.2013). Participants in the patients and control groups were informed about the purpose of the study, and verbal informed consent was obtained before enrollment in the study.

## Results

The results of the examination of blood serum in the patients and control groups are presented in Table 1.

No IgM-positive subjects were observed in our populations of patients and controls. In contrast, specific anti-*Toxoplasma* IgG antibodies were detected in 40% of those tested in the patients and in 25% of the control group. The absence of immunoglobulin IgM in conjunction with the presence of IgG suggests the presence of an exclusively latent form of toxoplasmosis in both the patients and control groups. The number of seropositive subjects with IgG in the patients was significantly higher than in the control group. Thus, among the patients with neuropsychiatric disorders, the incidence of cases with latent toxoplasmosis was more than twice than that in the control group.

The data in Table 1 show that the effect of toxoplasmosis was significant and similar for men (OR = 1.72, CI_95_ = 0.88±3.37, p<0.11) and women (OR = 2.19, CI_95_ = 1.08±4.43, p<0.03). A similar seropositive prevalence was found in men and women, in both in the patients and control groups, despite the fact that the prevalence of toxoplasmosis was approximately twice as high in the patients group. To assess the possible confounding effects of sex, we also performed the Mantel-Haenszel test. The OR adjusted for sex was 1.93, CI_95_ = 1.18±3.15.

## Discussion

The relationship between latent toxoplasmosis and mental disorders was well documented by Soviet researchers during the 1960s-1980s. The results of selected studies during the period of 1960–1970 are given in Table 2. The data relate to studies conducted in the territories of 2 Republics of the former USSR–Ukraine and the Russian Federation. All data were obtained through the use of Complement Fixation Test, only. Among various mental disorders, schizophrenia constituted between 50% and 89% [25].

**Table 2:** Toxoplasmosis in Mentally Ill Patients in the USSR (1964, 1966, 1970).

Source	Mental Disorders including Schizophrenia*		Controls	
	Examined (n)	Positive (%)	Examined (n)	Positive (%)
Anisimova [26]	24	19.2	178	5.0
Moiseeva [27]	858	27.9	128	7.0
Orestenko [28]	230	34.6	200	13.0
Savonenko and Karmanova [29]	224	9.2	227	0.0
Mihalev [25]	460	32.4	225	5.0
Petrov and Skokova [30]	202	31.1	183	9.2
Betin [31]	340	16.7	186	2.15
Motavkina et al. [32]	1504	10.1	160	2.6

*Number of schizophrenia cases greater than 50%

The data illustrated well established trend of the association between toxoplasmosis and schizophrenia in study areas of the USSR and it was in agreement with the results obtained in other countries abroad. It was shown that the seroprevalence of toxoplasmosis among patients with mental disorders was 2–3 times more frequent than among healthy persons [33, 34]. In Germany, the prevalence of toxoplasmosis in conjunction with various mental disorders was 43%-50% compared to 3% among healthy persons; in Poland, the prevalence was 51% compared to 25%; and in Czechoslovakia, the prevalence was 43% compared to 10%, respectively [33].

The results of our own studies demonstrate a considerable increase in toxoplasmosis prevalence both in the patients (40%) and, particularly, in the group of healthy individuals (25%) compared with the results from studies during the 1960s-1970s (Table 1).

Hypothetically, it could be interpreted as an expansion of toxoplasmosis among the population of the Russian Federation in parallel with the increase in mental disorders, particularly schizophrenia, during the following years. This hypothesis is supported by the official data from the period 1965–1987, when the number of mental outpatient cases, particularly schizophrenia cases, increased by 4.7-times [33].

A similar trend was observed during the following years after the dissolution of the USSR [35–38].

The results of our own studies are compatible with the data derived from similar studies in Germany (39% of schizophrenia patients were seropositive for toxoplasmosis) [39]. Similar data from other countries revealed that the prevalence of toxoplasmosis in schizophrenia patients varied from year to year and from place to place [40–43].

Meta-analysis of the results of 38 studies in different countries of the world revealed that aggregate OR was 2.73 [12], while it was considerably lower in our study at 1.93. This might be due to the vast proximity of the territory of the Russian Federation and the wide variation in the socioeconomic conditions of local people, resulting in a broad range of toxoplasmosis prevalence. For example, toxoplasmosis prevalence in the Tatarstan Republic at present is 31% [35], while it is 5%-9% in the Republic of Saha (northeast Siberia) [35] and approximately 10% in Omsk city (southern Siberia) [37].

The association between toxoplasmosis and schizophrenia and toxoplasmosis was found by hundreds of studies. However, the direct role of toxoplasmosis in etiology of schizophrenia is still sometimes questioned. In fact, until now, only one longitudinal study has examined the causality relation between toxoplasmosis and schizophrenia. Based on the criterion of temporality, it confirmed that the *Toxoplasma* infection is the cause, rather than the effect of schizophrenia [44].

Forty years ago, some researchers still believed that the high prevalence of toxoplasmosis among schizophrenia patients was related to poor sanitation and hygiene together with frequent contact with sources of infection [45]. The neurophysiological, pharmacological, genomic and epidemiological evidence accumulated during the past 30 years, however, proved beyond any reasonable doubt that the *Toxoplasma* infection is the cause, not the consequence of schizophrenia.

The probable role of *Toxoplasma* strains with different virulence in the etiology of mental diseases was studied in the USSR during the 1970s [46]. It was shown that virulent strains of *Toxoplasma* produce hyaluronidase, which facilitates the penetration of the parasite into host cells along with exotoxins with lethal and derma-necrotic properties; nonvirulent strains lack this enzyme. Furthermore, it was concluded that the lack of this enzyme is a biological characteristic of nonvirulent strains, facilitating their survival in the cyst form in the host for an extensive period of time.

A recently revised hypothesis on the important role of virulent strains of *Toxoplasma gondii* in the etiology of schizophrenia has received new attention. This hypothesis is based on the results of published data showing that virulent strains of *Toxoplasma gondii* are more frequently associated with the increased prevalence and severity of the clinical course of toxoplasmosis [47]. Moreover, evidence exists that certain drugs used for the treatment of schizophrenia inhibit replication of *T*. *gondii* [48].

The hypothesis takes also into account several factors probably contributing to the etiology of schizophrenia [14, 15, 49–53]. Importantly, hypothesis also considers that *T*.*gondii* might not be involved directly in the etiology of schizophrenia, but could interact with other genetic and/or environmental factors. Such possibility is well-tuned to Konovalov’s (1972) interpretation of the role of toxoplasmosis in mixed infections. It was demonstrated that in mixed infections involving toxoplasmosis, comembers of the parasitic system may possess both antagonistic and symbiotic characteristics, by which the severity of pathologic toxoplasmosis is determined. Activated toxoplasmosis infection aggravates other coinfections and diseases such as malignant tumors, tuberculosis, schizophrenia, etc.

It could also be beneficial to understand the degree of receptivity of the population towards contraction of infection. For example, the iso-antigens of the blood system ABO(H) could serve as markers of the latter. The results of the study in the Far East region of the Russian Federation indicated that women, carriers of iso-antigen A, and men, with iso-antigen AB, were more receptive to infection. Those carriers of iso-antigen B were relatively resistant to infection. The native population of Chukotka (Chukchi, Eskimos) is also less receptive [54].

### Strengths and limitations of the study

The strength of the study is seen in the provision the *Toxoplasma* seroprevalence data for underexplored region–Russian Federation. The limitation of the study is a relatively low number of participants and, in the absence of clinical and demographic data in respect of participants. The results of our studies might contribute to a better understanding of the reasons for the increasing incidence of schizophrenia in the Russian Federation. The association of latent toxoplasmosis and schizophrenia could be seen as one of the probable factors responsible for the disease. However, this study is limited in terms of the generalizability of the findings, as it examined the residents of Moscow City and the Moscow Region only.

## Conclusions

The results of our research are in agreement with the data obtained by Soviet scientists and their colleagues abroad during the 1960s-1980s with respect to trends of higher levels of antibodies to *Toxoplasma gondii* in patients with mental disorders, particularly schizophrenia, in comparison with healthy persons.

Our results are also compatible with the results of studies on the subject obtained in various parts of the world during the last 15–20 years. However, there is an appreciable difference (approximately 30%) regarding the OR index. It was 1.93 in our case as opposed to a cumulative OR of 2.73 in studies elsewhere. The reasons for this difference need further study.

Our publication should be considered as a resumption of studies carried out during the 1960s-1980s in the former USSR with respect to the association of latent toxoplasmosis and mental disorders, particularly schizophrenia. This is an important area of research in consideration of the heavy burden of disease in the Russian Federation.

## References

[1] Fond G, Capdevielle D, Macgregor A, Attal J, Larue A, Brittner M, et al *Toxoplasma gondii*: a potential role in the genesis of psychiatric disorders. L'Encephale. 2013;39(1):38–43. Epub 2012/10/26. 10.1016/j.encep.2012.06.014 . 23095600

[2] Tedford E, McConkey G. Neurophysiological changes induced by chronic *Toxoplasma gondii* Infection. Pathogens (Basel, Switzerland). 2017;6(2):19–31. 10.3390/pathogens6020019 . 28513566 PMC5488653

[3] Zasukhin D, Savina M. The role of protozoa in human and animal brain pathology (review of the literature). Zhurnal nevropatologii i psikhiatrii imeni SS Korsakova (Moscow, Russia: 1952). 1973;73(7):1083–8. 4204840

[4] Martin HL, Alsaady I, Howell G, Prandovszky E, Peers C, Robinson P, et al Effect of parasitic infection on dopamine biosynthesis in dopaminergic cells. Neuroscience. 2015;306:50–62. Epub 2015/08/25. 10.1016/j.neuroscience.2015.08.005 26297895 PMC4577654

[5] Siim JC. Clinical and diagnostic aspects of human acquired toxoplasmosis. Human Toxoplasmosis. Copenhagen: Ejna Munksgaard Forlag; 1960 p. 53–79.

[6] Flegr J, Escudero DQ. Impaired health status and increased incidence of diseases in *Toxoplasma*-seropositive subjects—an explorative cross-sectional study. Parasitology. 2016;143(14):1974–89. Epub 2016/10/11. 10.1017/S0031182016001785 . 27719690

[7] Hurley RA, Taber KH. Latent *Toxoplasmosis gondii*: emerging evidence for influences on neuropsychiatric disorders. The Journal of neuropsychiatry and clinical neurosciences. 2012;24(4):376–83. Epub 2012/12/12. 10.1176/appi.neuropsych.12100234 . 23224444

[8] Jones JL, Kruszon-Moran D, Wilson M, McQuillan G, Navin T, McAuley JB. *Toxoplasma gondii* infection in the United States: seroprevalence and risk factors. Am J Epidemiol. 2001;154(4):357–65. Epub 2001/08/10. 10.1093/aje/154.4.357 . 11495859

[9] Stepanova EV, Kondrashin AV, Sergiev VP, Morozova LF, Turbabina NA, Maksimova MS, et al Significance of chronic toxoplasmosis in epidemiology of road traffic accidents in Russian Federation. PloS one. 2017;12(9):e0184930 Epub 2017/09/29. 10.1371/journal.pone.0184930 28957427 PMC5619715

[10] Fabiani S, Pinto B, Bonuccelli U, Bruschi F. Neurobiological studies on the relationship between *Toxoplasmosis* and neuropsychiatric diseases. Journal of the neurological sciences. 2015;351(1–2):3–8. Epub 2015/03/03. 10.1016/j.jns.2015.02.028 . 25725931

[11] Flegr J, Hodný Z. Cat scratches, not bites, are associated with unipolar depression—cross-sectional study. Parasites & Vectors. 2016;9(1):8 10.1186/s13071-015-1290-7 26728406 PMC4700762

[12] Torrey EF, Bartko JJ, Lun ZR, Yolken RH. Antibodies to *Toxoplasma gondii* in patients with schizophrenia: a meta-analysis. Schizophrenia bulletin. 2007;33(3):729–36. Epub 2006/11/07. 10.1093/schbul/sbl050 17085743 PMC2526143

[13] Torrey EF, Bartko JJ, Yolken RH. *Toxoplasma gondii* and other risk factors for schizophrenia: an update. Schizophr Bull. 2012;38(3):642–7. Epub 2012/03/27. 10.1093/schbul/sbs043 22446566 PMC3329973

[14] Flegr J, Preiss M, Klose J, Havlicek J, Vitakova M, Kodym P. Decreased level of psychobiological factor novelty seeking and lower intelligence in men latently infected with the protozoan parasite *Toxoplasma gondii* Dopamine, a missing link between schizophrenia and toxoplasmosis? Biol Psychol. 2003;63(3):253–68. Epub 2003/07/11. . 12853170 10.1016/s0301-0511(03)00075-9

[15] Skallovà A, Novotna M, Kolbekova P, Gasova Z, Vesely V, Sechovska M, et al Decreased level of novelty seeking in blood donors infected with *Toxoplasma*. Neuro Endocrinol Lett. 2005;26(5):480–6. Epub 2005/11/03. . 16264415

[16] Flegr J. Influence of latent *Toxoplasma* infection on human personality, physiology and morphology: pros and cons of the *Toxoplasma*-human model in studying the manipulation hypothesis. The Journal of experimental biology. 2013;216(Pt 1):127–33. Epub 2012/12/12. 10.1242/jeb.073635 . 23225875

[17] Ziryanov SK, Belousov DY, Afanasieva EV, Efremova EA. Cost-effectiveness analysis of the use of modern anti-psychotic drugs in schizophrenia patients. Qualitative Clinical Practice. 2013;2:18–23.

[18] Schizophrenia. Available from: https://www.who.int/en/news-room/fact-sheets/detail/schizophrenia.

[19] *Mental Health Atlas-2017* 2017; Available from: https://www.who.int/mental_health/evidence/atlas/profiles-2017/en/.

[20] Sivukha T, Shevkunova E. Diagnosis of nervous system lesions in toxoplasmosis. Zhurnal nevropatologii i psikhiatrii imeni SS Korsakova (Moscow, Russia: 1952). 1969;69(10):1464–5. 5369939

[21] Zasukhin DN, Shevkunova EA. The main results of studies on toxoplasmosis in the USSR. Med Parazitol. 1967;36(6):695–701. Epub 1967/11/01. . 5611213

[22] Khodorkovsky AV. Schizophrenia. Available from: www.medicalj.ru/diseases/psychiatrics/112-schizophrenia.

[23] Lubov EB, Yastrebov VS, Schevchenko LS, Chapurin SA. Economic burden of schizophrenia in Russia. Social and Clinical Psychiatry. 2012;22(3):3–36.

[24] Centers for Disease Control and Prevention. Epi info™ for windows. Available from: https://www.cdc.gov/epiinfo/pc.html.

[25] Mihalev PV. On neuro-psychiatric disorders in toxoplasmosis and efficacy of treatment Toxoplasmosis. Kiev: Health; 1966 p. 21–3.

[26] Anisimova AI. Preliminary materials on distribution of toxoplasmosis in the Transcarpatian region Toxoplasmosis. Kiev: Health; 1964 p. 15–7.

[27] Moiseeva KV. Laboratory and epidemiological data on toxoplasmosis in Herson region Toxoplasmosis. Kiev: Health; 1964 p. 18–20.

[28] Orestenko LP. Toxoplasmosis in miners in Lugansk region Toxoplasmosis. Kiev: Health; 1966 p. 21–2.

[29] Savonenko EA, Karmanova EV. Toxoplasmosis in the far East of Russia Toxoplasmosis. Kiev: Health; 1966 p. 45–8.

[30] Petrov VP, Skokova LP. On toxoplamosis problem in Kuibishev region Toxoplasmosis. Kiev: Health; 1966 p. 59–63.

[31] Betin EM. Materials on toxoplasmosis in patients with mental disorders. Korsakov Journal Neurology Psychiatry. 1969;69(6):909–13. 5370146

[32] Motavkina NS, Mikhaleva LV, Kotkov FI, Fraĭnd NM. The immunologic reactivity of mental patients with toxoplasmosis. Zhurnal nevropatologii i psikhiatrii imeni SS Korsakova (Moscow, Russia: 1952). 1970;70(5):718–21. 5451865

[33] Khaletskii AM, Zasukhin DN. Toxoplasmosis and neuropsychiatric disorders; review of literature. Zhurnal nevropatologii i psikhiatrii imeni SS Korsakova (Moscow, Russia: 1952). 1956;56(5):405–9. 13353738

[34] Korovitski LK. Classification of clinical manifestations of toxoplasmosis Toxoplasmosis. Kiev: Health; 1964 p. 145–52.

[35] Shuralev EA, Shamaev ND, Mukminov MN, Nagamune K, Taniguchi Y, Saito T, et al *Toxoplasma gondii* seroprevalence in goats, cats and humans in Russia. Parasitol Int. 2018;67(2):112–4. 10.1016/j.parint.2017.10.014 29126978

[36] Magnaval JF, Leparc-Goffart I, Gibert M, Gurieva A, Outreville J, Dyachkovskaya P, et al A serological survey about zoonoses in the Verkhoyansk area, Northeastern Siberia (Sakha Republic, Russian Federation). Vector borne and zoonotic diseases (Larchmont, NY). 2016;16(2):103–9. Epub 2016/01/26. 10.1089/vbz.2015.1828 . 26807914

[37] Dolgikh TI, Zaparii NS, Kadtsyna TV, Kalitin AV. Epidemiological and clinicoimmunological monitoring of *Toxoplasmosis* in the Omsk region. Med Parazitol. 2008;(1):19–22. Epub 2008/03/28. . 18365468

[38] Zharikov NM, Kiselev AS. Psychiatric services in the USSR and their various indicators. Zhurnal nevropatologii i psikhiatrii imeni SS Korsakova (Moscow, Russia: 1952). 1990;90(11):70–4. Epub 1990/01/01. . 1963986

[39] Hinze-Selch D, Daubener W, Eggert L, Erdag S, Stoltenberg R, Wilms S. A controlled prospective study of *Toxoplasma* gondii infection in individuals with schizophrenia: beyond seroprevalence. Schizophrenia bulletin. 2007;33(3):782–8. Epub 2007/03/28. 10.1093/schbul/sbm010 17387159 PMC2526145

[40] Cetinkaya Z, Yazar S, Gecici O, Namli MN. Anti-*Toxoplasma gondii* antibodies in patients with schizophrenia—preliminary findings in a Turkish sample. Schizophrenia bulletin. 2007;33(3):789–91. Epub 04/02. 10.1093/schbul/sbm021 . 17404388 PMC2526136

[41] Xu X, Sun F, Chao H, Qian Y, Chen J, Sun M. Investigation and study of sero-epidemiology on *Toxoplasma gondii* infection in special population. Re Dai Yi Xue. 2005;3:133–6.

[42] Yuksel P, Alpay N, Babur C, Bayar R, Saribas S, Karakose AR, et al The role of latent toxoplasmosis in the aetiopathogenesis of schizophrenia—the risk factor or an indication of a contact with cat? Folia Parasitol. 2010;57(2):121–8. Epub 2010/07/09. . 20608474 10.14411/fp.2010.015

[43] Zhu S, Lin Y, Wang S, Xu S. Contrast study on schizophrenia’s toxoplasmosis infection rate. Med J Chin People Health. 2003;15:405–7.

[44] Niebuhr DW, Millikan AM, Cowan DN, Yolken R, Li Y, Weber NS. Selected infectious agents and risk of schizophrenia among U.S. military personnel. The American journal of psychiatry. 2008;165(1):99–106. Epub 2007/12/19. 10.1176/appi.ajp.2007.06081254 . 18086751

[45] Delgado GG. Toxoplasmosis and mental diseases. Revista cubana de medicina tropical. 1979;31(2):127–31. Epub 1979/05/01. . 395584

[46] Konovalov SI. Biological peculiarities of Toxoplasma, circulation in the environment and immunity aspects. Dissertation, Doctor of science, Alma-Ata 1972.

[47] Xiao J, Yolken RH. Strain hypothesis of *Toxoplasma gondii* infection on the outcome of human diseases. Acta Physiol. 2015;213(4):818–45. Epub 2015/01/21. 10.1111/apha.12458 25600911 PMC4361247

[48] Jones-Brando L, Torrey EF, Yolken R. Drugs used in the treatment of schizophrenia and bipolar disorder inhibit the replication of *Toxoplasma gondii*. Schizophrenia research. 2003;62(3):237–44. Epub 2003/07/03. . 12837520 10.1016/s0920-9964(02)00357-2

[49] Xiao J, Li Y, Prandovszky E, Karuppagounder SS, Talbot CC, Dawson VL, et al MicroRNA-132 dysregulation in *Toxoplasma gondii* infection has implications for dopamine signaling pathway. Neuroscience. 2014;268:128–38. Epub 03/18. 10.1016/j.neuroscience.2014.03.015 . 24657774 PMC4085776

[50] Fuks JM, Arrighi RB, Weidner JM, Kumar Mendu S, Jin Z, Wallin RP, et al GABAergic signaling is linked to a hypermigratory phenotype in dendritic cells infected by *Toxoplasma gondii*. PLoS pathogens. 2012;8(12):e1003051 Epub 2012/12/14. 10.1371/journal.ppat.1003051 23236276 PMC3516538

[51] Schwarcz R, Hunter CA. *Toxoplasma gondii* and schizophrenia: linkage through astrocyte-derived kynurenic acid? Schizophrenia bulletin. 2007;33(3):652–3. Epub 2007/04/17. 10.1093/schbul/sbm030 17434932 PMC2526138

[52] Prandovszky E, Gaskell E, Martin H, Dubey JP, Webster JP, McConkey GA. The neurotropic parasite *Toxoplasma gondii* increases dopamine metabolism. PLoS One. 2011;6(9):e23866 Epub 2011/10/01. 10.1371/journal.pone.0023866 21957440 PMC3177840

[53] Betin EM. Diagnosis of Toxoplasmosis infection of the brain with epileptiform syndrome. Med Parazitol. 1970;39(4):416–21. Epub 1970/07/01. . 5488530

[54] Yakovlev AA. Epidemiological and immunological aspects of studies on toxoplasmosis in the far East of Russia, Dissertation, PhD. Leningrad1980.

